# Unraveling intestinal microbiota’s dominance in polycystic ovary syndrome pathogenesis over vaginal microbiota

**DOI:** 10.3389/fcimb.2024.1364097

**Published:** 2024-03-28

**Authors:** Xia Yu, XiaoQin Li, Hui Yang

**Affiliations:** Hunan Women and Children’s Hospital, Changsha, China

**Keywords:** polycystic ovary syndrome, intestinal microbiota, vaginal microbiota, sex hormone, dehydroepiandrosterone

## Abstract

**Background:**

Polycystic ovary syndrome (PCOS) is a prevalent endocrine disease in women, intricately linked to hormonal imbalances. The microbiota composition plays a pivotal role in influencing hormonal levels within the body. In this study, we utilized a murine model to investigate how intestinal and vaginal microbiota interact with hormones in the development of PCOS.

**Methods:**

Twenty female mice were randomly assigned to the normal group (N) and the model group (P), where the latter received daily subcutaneous injections of 0.1 mL DHEA (6 mg/100 g). Throughout the experiment, we evaluated the PCOS mouse model by estrus cycle, serum total testosterone (T), prolactin (PRL) and luteinizing hormone (LH) levels, and ovarian pathological morphology. The microbial composition in both intestinal content and vaginal microbiota were studied by 16S rRNA gene high-throughput sequencing.

**Results:**

Compared with the N group, the P group showed significant increases in body weight, T, and PRL, with significant decrease in LH. Ovaries exhibited polycystic changes, and the estrous cycle was disrupted. The intestinal microbiota result shows that Chao1, ACE, Shannon and Simpson indexes were decreased, Desulfobacterota and Acidobacteriota were increased, and *Muribaculaceae*, *Limosilactobacillus* and *Lactobacillus* were decreased in the P group. T was significantly positively correlated with *Enterorhabdus*, and LH was significantly positively correlated with *Lactobacillus.* The analysis of vaginal microbiota revealed no significant changes in Chao1, ACE, Shannon, and Simpson indices. However, there were increased in Firmicutes, Bacteroidota, Actinobacteriota, *Streptococcus*, and *Muribaculaceae*. Particularly, *Rodentibacter* displayed a robust negative correlation with other components of the vaginal microbiota.

**Conclusion:**

Therefore, the response of the intestinal microbiota to PCOS is more significant than that of the vaginal microbiota. The intestinal microbiota is likely involved in the development of PCOS through its participation in hormonal regulation.

## 1 Introduction

Polycystic ovary syndrome (PCOS) is characterized by irregular menstrual cycles, increased body hair, ovulatory dysfunction (OD), hyperandrogenemia (HA), and polycystic ovarian morphology (PCOM) (Rosenfield, 2020; Chen et al., 2022). As of 2020, the prevalence of PCOS has reached 26% (Wolf et al., 2018; Zeng et al., 2022), making it a significant reproductive endocrine disorder that profoundly impacts the fertility of women in their childbearing years (Zhu and Goodarzi, 2022). Sex hormone dysregulation stands out as a pivotal pathological mechanism in PCOS. Excessive androgens in the ovaries are recognized as the primary culprits in the development of PCOS. Beyond their detrimental effects on ovarian function, these excess androgens also disrupt the follicular microenvironment, leading to follicular atresia in affected individuals (Rosenfield and Ehrmann, 2016; Zeng et al., 2020). Consequently, the utilization of anti-androgen medications has emerged as a principal therapeutic approach. Changes in sex hormone levels are intricately linked to modifications in both the intestinal and vaginal microbiota, playing a pivotal role in the intestinal-vaginal microbiota axis. Fluctuations in sex hormones contribute to gender dimorphism in the intestinal microbiota (Wu et al., 2022). Estrogen levels undergo regular fluctuations, shaping alterations in the intestinal microbiota during various life stages (Zhang et al., 2021). This interplay regulates immune response processes impacting women’s reproductive health. The intestinal microbiota influences circulating estrogens, shaping the vaginal microbiota and promoting reproductive tract health (Graham et al., 2021; Pace and Watnick, 2021). Androgens are synthesized or catabolized by the intestinal microbiota, influencing disease onset and progression (Li D. et al., 2022; Hsiao et al., 2023).

A large number of studies have found that intestinal flora and its metabolites have the potential to regulate the hypothalamic-pituitary axis and luteinizing hormone secretion through the gut axis, and that the imbalance of intestinal microbiota is associated with HA, insulin resistance, chronic inflammation, and metabolic disorders related to PCOS (Guo et al., 2023; Sun et al., 2024). Studies reveal a reduced relative abundance of *Lactobacillus*, *Ruminococcus*, and *Clostridium*, coupled with elevated *Prevotella* levels, in the intestines of PCOS-afflicted rats (Guo et al., 2016). Additionally, PCOS is hypothesized to manifest reduced intestinal bacterial diversity and an altered Firmicutes/Bacteroidota (F/B) ratio (Thackray, 2019). The vaginal microbiota, crucial for maintaining female reproductive health, actively regulates vaginal pH, provides protection against infections, and fortifies the immune system. Clinical investigations unveiled an increase in pathogens such as *Mycoplasma* and *Prevotella*, alongside a reduction in *Lactobacillus* levels in the vaginas and cervixes of affected individuals (Hong et al., 2020; Tu et al., 2020). Consequently, research into the microbiota of the intestines and vagina underscores their critical roles in women’s reproductive health.

Our study aimed to establish and assess a PCOS mouse model, investigating changes in both intestinal and vaginal microbiota by 16S rRNA gene sequencing. Simultaneously, we measured serum levels of total testosterone (T), prolactin (PRL), and luteinizing hormone (LH), and employed correlation networks to explore the interaction between microbiota and sex hormones in PCOS pathogenesis. Our study will pave the way for targeted therapeutic strategies to enhance PCOS management by addressing both intestinal and vaginal microbiota.

## 2 Materials and methods

### 2.1 Animals

In order to detect the estrous cycle of mice, twenty 3-week-old SPF-grade female Kunming mice were used in this study. The animals were purchased from Hunan Slacks Kingda Laboratory Animal Co. Ltd. and feed in the Laboratory Animal Center of Hunan University of Chinese Medicine (Changsha, China) under the following conditions: temperature of 23-25°C, humidity of 47-53%, 12-hour light/12-hour dark cycle, and free diet and water.

### 2.2 Drugs and reagents

**Table 1** outlines the key reagents, drugs, and kits employed in the study. Dehydroepiandrosterone (DHEA) was meticulously measured at 6 mg/100 g. The dissolution of DHEA involved the use of a solvent blend comprising 0.09 mL sesame oil and 0.01 mL ethanol (95%) to yield the DHEA injection.

**Table 1:** The key reagents, drugs, and kit.

Name	Manufacturer	Batch number
DHEA	Shanghai Macklin Biochemical Co., Ltd	C15163002
sesame oil	Beijing Solarbio Science Technology Co.,Ltd	331G025
95% ethanol	ANNJET Technology Co.,Ltd	20230602
1% Crystalline Violet	Beijing Solarbio Science Technology Co.,Ltd	G1063
T ELISA test kit	Jingmei Biotechnology Co.,Ltd	JM02836M2
PRL ELISAtest kit	Jingmei Biotechnology Co.,Ltd	JM02852M2
LH ELISAtest kit	Jingmei Biotechnology Co.,Ltd	JM11607M2
Isoflurane	Shandong Ante Animal Husbandry Technology Co., Ltd.	2023052902

### 2.3 Animal grouping and modeling

After 3 days of acclimatization feeding, mice were divided into the normal (N) and model (P) groups according to a random number generation table. According to the references, mice in the P group were injected subcutaneously with 0.1 mL of DHEA injection (DHEA content of 6 mg/100 g) every day, while mice in the N group were injected with 0.1 mL of saline for 21 consecutive d (Dou et al., 2018; Han et al., 2022). The mice were sterilized with 75% alcohol before and after each injection. During the modeling period, the body mass of the mice was measured weekly and the dose of DHEA was adjusted according to the weekly change in body weight.

### 2.4 Model evaluation

The criteria for evaluating the mouse PCOS model were established in accordance with the 2003 Rotterdam Criteria and the Expert Consensus on the Diagnosis and Treatment of PCOS by the Chinese Medical Association (Lee et al., 1991; Rotterdam ESHRE/ASRM-Sponsored PCOS consensus workshop group, 2004). Diagnosis of PCOS necessitated meeting at least two of the following criteria: (1) HA: The main manifestations are hirsutism, acne and hair loss, elevated androgens such as T, androstenedione, (2) OD: The main manifestations are oligomenorrhea or amenorrhea, elevated serum PRL and decreased LH, and disorders of the estrous cycle. (3) PCOM: Lack of follicles > 10 mm in diameter in follicular phase ovaries, ≥ 12 follicles 2 ~ 9 mm in diameter in one or both ovaries.

### 2.5 Vaginal smear

Starting from the 11th day of the PCOS modeling, daily morning assessments of vaginal swab smears were conducted. A sterile lance tip containing 10 -20 µL of sterile saline was gently inserted into the vagina of the mice, and the saline was pumped into the vagina and then aspirated out, repeating 2 -3 times. Finally, 10 ul of vaginal douche solution was dripped on the slide, dried naturally and fixed with 95% ethanol for 10 min, stained using 0.1% crystal violet for 1 min, rinsed with running water for 30s and dried. The slides were placed under an ordinary light microscope for cytological observation of vaginal epithelial cell morphology and photographed. According to the characteristics of the cell changes in the vaginal smears, they were classified as pre-estrus, estrus, post-estrus and inter-estrus (Han et al., 2022).

### 2.6 Detection of T, PRL and LH levels in serum

After the molding, mice were anesthetized through isoflurane inhalation, and blood samples were obtained from the eyeballs. Subsequently, the collected blood samples were allowed to stand at 4°C for a duration of 2 hours. The supernatant was carefully aspirated into Ep tubes and subjected to centrifugation in a low-temperature, high-speed centrifuge at 3000 r/min for 10 minutes, the upper layer of the serum was taken, and the samples were spiked according to the instructions of the ELISA kit. The serum samples were detected for the levels of T, PRL, and LH using the enzyme-labeling analyzer.

### 2.7 Histopathologic sections of the ovary

The ovaries of mice were dissected and removed under aseptic conditions, fixed in 4% paraformaldehyde solution and stored at room temperature. According to gradient dehydration, paraffin embedding after xylene transparency, made into 5um sections, the mouse ovary tissue sections were routinely deparaffinized, stained with hematoxylin and eosin (HE) solution sequentially, sealed with neutral gum, and photographed under the microscope for observation (Liu et al., 2023; Zhou et al., 2024).

### 2.8 Sample collection

After 21 days, the mice were executed by cervical dislocation and the small intestines of the mice were immediately removed by opening the abdominal cavity on an ultra-clean bench. Under aseptic conditions, the intestinal tissue from the gastric pylorus to the ileocecal region was incised longitudinally with sterile scissors, and the intestinal contents were clipped with sterile forceps, and the intestinal contents of each mouse were collected in an Ep tube and stored at -80°C (Qiao et al., 2023).

After disinfecting the vulva, a sterile cotton swab was used to swab the secretion from the cervical opening without touching the vaginal wall, rolling in a circular motion 2-3 times to obtain a full sample, and the head of the swab was cut off with sterile scissors, and the head of the swab was put into a sterile Ep tube for sealing; 3 swabs were repeated for each sample, and the samples were placed in the refrigerator at -80°C for freezing and preservation after taking the samples (Malaluang et al., 2024).

### 2.9 DNA extraction, 16S rRNA gene amplicon sequencing, and sequence analysis

All samples were processed by Shanghai Personal Biotechnology Co., Ltd (Shanghai, China), and the total microbial genomic DNA from each tube was extracted following the procedure for extracting nucleic acid instructions from the Omega Soil DNA Kit (D5625-01) kit (Omega Bio-Tek, Norcross, GA, USA). The quantity and quality of extracted DNAs were measured using a NanoDrop NC2000 spectrophotometer (Thermo Fisher Scientific, Waltham, MA, USA) and agarose gel electrophoresis, respectively. PCR amplification of the bacterial 16S rRNA genes V3–V4 region was performed using the forward primer 338F (5’-ACTCCTACGGGAGGCAGCA-3’) and the reverse primer 806R (5’-GGACTACHVGGGTWTCTAAT-3’). PCR products were quantified after purification of PCR amplicons and then mixed according to the amount of data required for each sample. Finally, the library was constructed using Illumina’s TruSeq Nano DNA LT Library Prep Kit, and 2×250bp bipartite sequencing was performed on an Illumina NovaSeq machine using the NovaSeq 6000 SP Reagent Kit (500 cycles) (Li C. et al., 2022).

### 2.10 Bioinformatics

To improve the quality of analytical results, the adequacy and quality of sequences be guaranteed. Therefore, the original sequence data were modified (QIIIME2), cut, filtered, de-noised, merged, and chimeric removed (DADA2) to obtain effective sequences before analysis. Sequence data analyses were mainly performed using QIIME2 and R packages (v3.2.0). ASV-level alpha diversity indices, such as Chao1 richness estimator, Observed species, Shannon diversity index, and Simpson index. ASV-level ranked abundance curves were generated to compare the richness and evenness of ASVs among samples. Beta diversity analysis was performed to investigate the structural variation of microbial communities across samples using Bray-Curtis metrics and UniFrac distance metrics and visualized via principal coordinate analysis (PCoA) was also conducted based on the genus-level compositional profiles. According to the taxonomy, the community structure was analyzed statistically at different taxonomic levels. For linear discriminant effect size (LefSe), the Kruskal-Wallis rank sum test and Wilcoxon rank sum test were performed, followed by linear discriminant analysis (LDA) to assess the effect size of each differential abundance taxa. Based on the above analysis, in-depth statistical and visual analysis of community structure and system development can be carried out (Qiao et al., 2023).

### 2.11 Statistical analysis

SPSS 25.00 software was used for statistical analysis, Quantitative data in each group were expressed as mean ± standard deviation. Independent samples t-test was used if the two groups of data conformed to normal distribution with chi-square. If the data did not conform to a normal distribution with chi-square, the Mann-Whitney U test was used. *P* < 0.05 indicates a statistically significant difference.

## 3 Results

### 3.1 Model evaluation

#### 3.1.1 Body weight changes and estrous cycle evaluation in modeled mice

**Figure 1:**
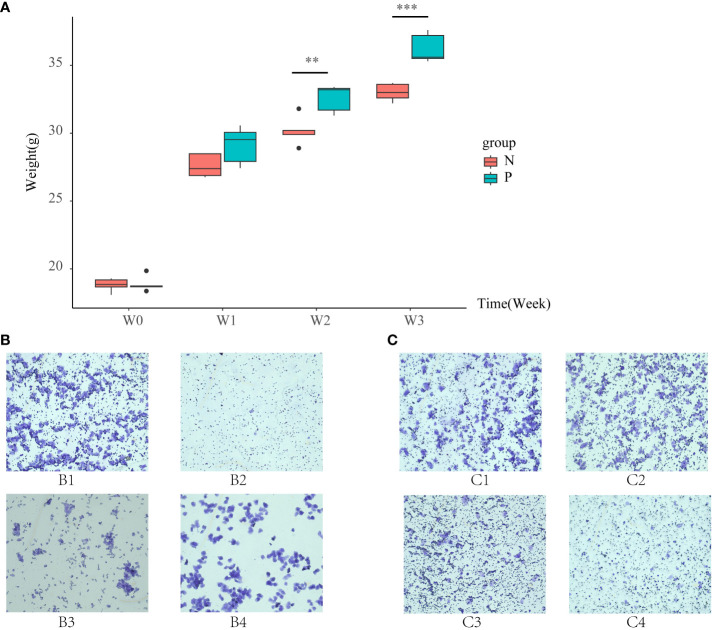
**(A)** Box line plots illustrating the changes in body weight of mice in the N group and the P group, **(B)** vaginal smears of mice in the N group, and **(C)** vaginal smears of mice in the P group (N: the normal group, P: the model group). **P<0.01, ***P<0.01.

Before modeling, the body weights of the N and P groups were similar. After 21 days of modeling, the body weight of mice in the P group was significantly higher than that of the N group (*P* < 0.05, as shown in **Figure 1A**). The estrous cycle of mice in the N group was normal. In the late estrous stage, irregular keratinized epithelial cells, nucleated epithelial cells, and leukocytes were all visible (**Figure 1**-B1). During the estrous interval, leukocytes were predominant, with occasional nucleated epithelial cells (**Figure 1**-B2). Proestrus showed a significant presence of oval-shaped nucleated epithelial cells, with occasional small numbers of leukocytes and keratinized epithelial cells (**Figure 1**-B3). Estrus was characterized by irregularly shaped keratinized epithelial cells arranged in a deciduous pattern, with a minimal number of leukocytes and nucleated epithelial cells visible (**Figure 1**-B4). In contrast, the estrous cycle of mice in the P group was delayed, primarily observed in the estrous interval and late estrous stage (**Figure 1C**).

#### 3.1.2 Changes in ovarian tissue morphology and sex hormones after modeling

**Figure 2:**
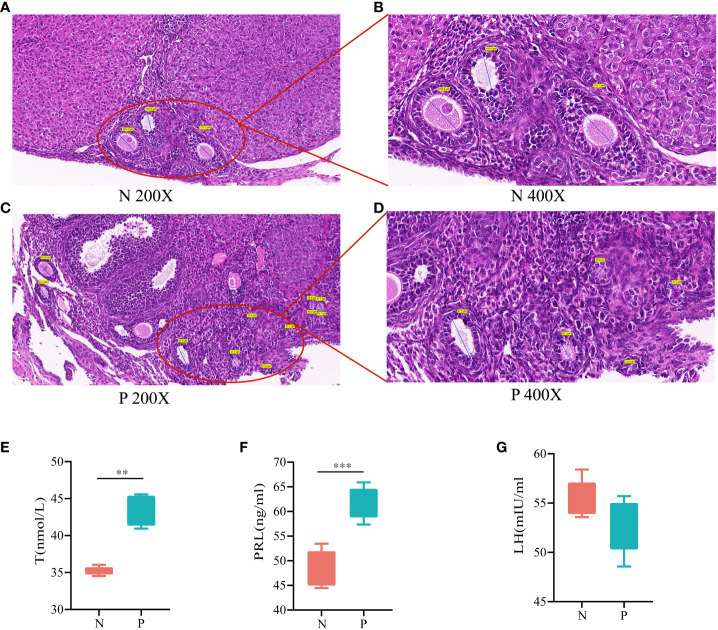
**(A)** HE staining of ovarian tissues in the N group (200X), **(B)** HE staining of ovarian tissues in the N group (400X), **(C)** HE staining of ovarian tissues in the P group (200X), **(D)** HE staining of ovarian tissues in the P group (400X), **(E)** serum levels of T, **(F)** serum levels of PRL, and **(G)** serum levels of LH (N, the normal group; P, the model group). **P<0.01,***P<0.01.

Histopathologic sections of the ovaries showed multiple follicles larger than 10 mm in diameter in the ovaries during the follicular stage in the N group (**Figures 2A, B**). In contrast, during the follicular stage in the P group, although follicles larger than 10 mm in diameter were present, there were≥12 follicles ranging from 2 to 9 mm in diameter in one ovary, indicative of polycystic changes in the ovaries (**Figures 2C, D**). T was significantly higher in the P group than in the N group (**Figure 2E**, *P*<0.05), consistent with HA. PRL was significantly higher in the P group than in the N group (**Figure 2F**, *P*<0.05) while LH was lower than in the N group (**Figure 2G**, *P*>0.05), which, combined with the vaginal smears of the mice, were consistent with the diagnosis of abnormal ovulation. In conclusion, mice in the P group met the diagnostic criteria for PCOS, confirming the success of the model replication.

### 3.2 Changes in intestinal microbiota and vaginal microbiota in PCOS mice

#### 3.2.1 Changes in the diversity of intestinal microbiota and vaginal microbiota in PCOS mice

**Figure 3:**
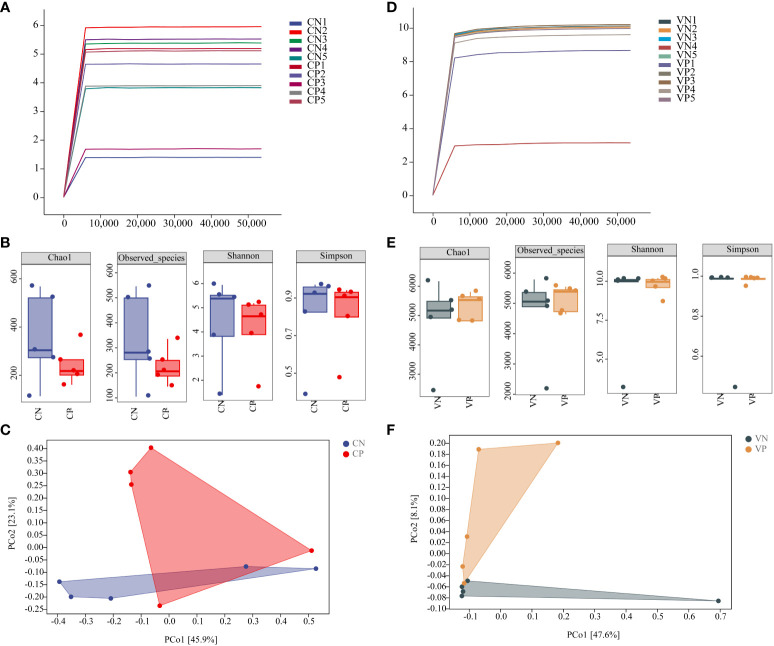
**(A)** Shannon dilution curve of mouse intestinal microbiota, **(B)** Alpha diversity of mouse intestinal microbiota, **(C)** PCoA analysis of mouse intestinal microbiota based on the Bray-Curtis distance algorithm, **(D)** Shannon dilution curve of mouse vaginal microbiota, **(E)** Alpha diversity of mouse vaginal microbiota, and **(F)** PCoA analysis of mouse vaginal microbiota based on the Bray-Curtis distance algorithm for PCoA analysis of mouse vaginal microbiota (CN, intestinal microbiota in the normal group; CP, intestinal microbiota in the model group; VN, vaginal microbiota in the normal group; VP, vaginal microbiota in the model group).

The Shannon dilution curves for intestinal contents microbiota and vaginal microbiota showed a subsequent decrease in the growth rate of the number of ASVs as the number of sequencing increased, suggesting that the amount of sequencing data was sufficient for the present analyses (**Figures 3A, D**). Alpha diversity reflects species abundance and species diversity of individual samples, where Chao1 and ACE indices measure species abundance and Shannon and Simpson indices are used to measure species diversity. Compared to the CN group, the Chao1, ACE, Shannon, and Simpson indices in the CP group exhibited a decreasing trend (*P* > 0.05, **Figure 3B**). Similarly, when compared to the VN group, the Chao1, ACE, Shannon, and Simpson indices in the VP group did not show significant changes (**Figure 3E**). Beta diversity, evaluated using the Bray-Curtis distance algorithm, revealed in PCoA analysis that the CN group was more distant from the CP group, and the VN group was more distant from the VP group, indicating substantial group differences (**Figure 3C**, **F**). Therefore, the results of this part of the experiment showed that the bacterial microbiota of the mice after modeling showed a significant separation from the N group, indicating that some changes in the bacterial microbiota were found, in which the diversity of the intestinal microbiota of the PCOS mice showed a decreasing trend.

#### 3.2.2 Altered composition of intestinal and vaginal microbiota in PCOS mice

**Figure 4:**
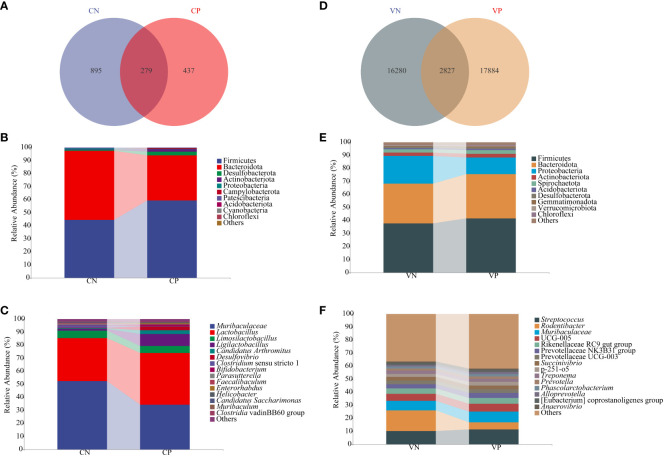
**(A)** ASV count of mouse intestinal microbiota, **(B)** Relative abundance of mouse intestinal microbiota at the phylum level **(C)** Relative abundance of mouse intestinal microbiota at the genus level, **(D)** ASV count of mouse vaginal microbiota, **(E)** Relative abundance of mouse vaginal microbiota at the phylum level, and **(F)** Relative abundance of mouse vaginal microbiota at the genus level (CN, intestinal microbiota in the normal group; CP, intestinal microbiota in the model group; VN, vaginal microbiota in the normal group; VP, vaginal microbiota in the model group).

Sequences exhibiting similarity higher than 100% were consolidated into a single AVS cluster, resulting in 1,174 ASV in the CN group and a total of 716 ASV in the CP group, with 279 identical ASV shared between the two groups (**Figure 4A**). In the VN group, there were 19,107 ASV, and a total of 20,711 ASV were identified in the VP group, with 2,827 identical ASV between the two groups (**Figure 4D**). To discern bacteria associated with PCOS progression, we conducted an analysis of the composition and variation of the intestinal content microbiota and vaginal microbiota, focusing on the top 10 relative abundances at the phylum level and the top 15 relative abundances at the genus level.

At the phylum level, the microbiota of the intestinal contents in each group, as illustrated in **Figure 4B**, were primarily composed of Firmicutes, Bacteroidota, Desulfobacteriota, Actinobacteriota, and Proteobacteria. In comparison to the CN group, the CP group exhibited elevated levels of Firmicutes and Desulfobacteriota (*P* > 0.05), while Actinobacteriota showed a significant increase (*P* < 0.05), and Bacteroidota and Proteobacteria decreased (*P* > 0.05). At the genus level, as depicted in **Figure 4C**, the microbiota of the intestinal contents in each group was dominated by *Muribaculaceae*, *Lactobacillus*, *Limosilactobacillus*, *Ligilactobacillus*, and *Candidatus Arthromitus*. In the CP group, relative to the CN group, *Muribaculaceae* and *Limosilactobacillus* decreased, while *Lactobacillus*, *Ligilactobacillus*, and *Candidatus Arthromitus* increased (*P* > 0.05).

As illustrated in **Figure 4E**, the dominant phyla of the vaginal microbiota in mice in each group were Firmicutes, Bacteroidota, Proteobacteria, Actinobacteriota, and Spirochaetota. In comparison with the VN group, the VP group displayed higher levels of Firmicutes and elevated Bacteroidota and Actinobacteriota, while Proteobacteria decreased in the VP group compared to the VN group (*P* > 0.05). Shown in **Figure 4F**, the dominant genera of vaginal microbiota in mice in each group were *Streptococcus*, *Rodentibacter*, and *Muribaculaceae*. *Streptococcus* and *Muribaculaceae* were elevated in the VP group, while *Rodentibacter* decreased compared with the VN group (*P* > 0.05).

In summary, PCOS mice displayed a reduced count of intestinal microbiota ASV and an increased count of vaginal microbiota ASV. The community composition of the P group and the N group was quite different. This study showed that after modeling, the overall abundance and bacterial diversity of intestinal microbiota in PCOS mice showed a downward trend, Firmicutes, Bacteroidota and Actinobacteriota in the VP group increased.

#### 3.2.3 Characteristic bacteria of intestinal and vaginal microbiota in PCOS mice

**Figure 5:**
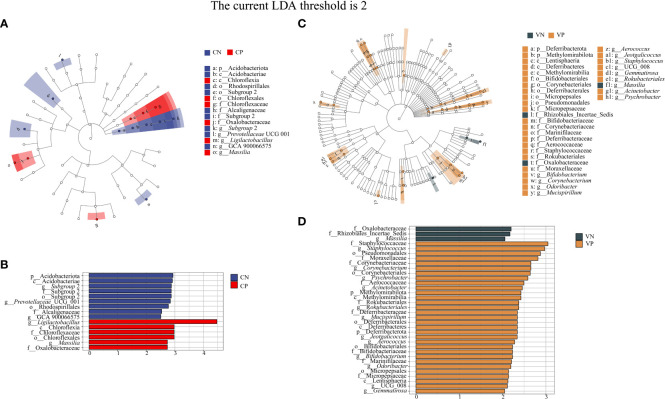
**(A)** Evolutionary branching diagram of LEfSe analysis of mouse intestinal microbiota, **(B)** Histogram of LDA value distribution of mouse intestinal microbiota, **(C)** Evolutionary branching diagram of LEfSe analysis of mouse vaginal microbiota, **(D)** Histogram of LDA value distribution of mouse vaginal microbiota (The current LDA threshold is 2, CN, intestinal microbiota in the normal group; CP, intestinal microbiota in the model group; VN, vaginal microbiota in the normal group; VP, vaginal microbiota in the model group).

Based on LEfSe linear discriminant analysis, we identified differences in microbiota abundance between the CN, CP, VN and VP groups, with multiple bacteria identified as key discriminators. When LDA = 2, *Subgroup* 2, *Prevotellaceae* UCG 001 and GCA 900066575 were significantly enriched in the CN group, and *Ligilactobacillus* and *Massilia* were significantly enriched in the CP group (**Figures 5A, B**). *Massilia* was significantly enriched in the VN group and *Bifidobaterium*, *Corynebacterium*, *Odoribacter*, *Mucispirillum*, *Aerococcus*, *Jeotgalicoccus*, *Staphylococcus*, UCG 008, *Gemmatirosa*, *Rokubacteriales*, *Acinetobacter*, and *Psychrobacter* were significantly enriched in the VP group (**Figures 5C, D**).

#### 3.2.4 Altered metabolic functions of intestinal and vaginal microbiota in PCOS mice

**Figure 6:**
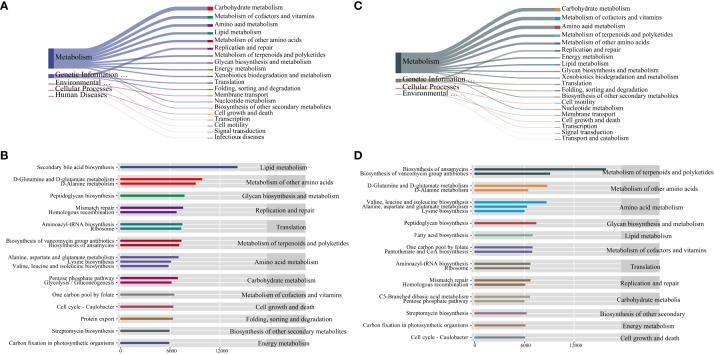
**(A)** Sankey diagram of primary and secondary metabolic pathways of intestinal microbiota, **(B)** Histogram of horizontal distribution of secondary and tertiary metabolic pathways of intestinal microbiota, **(C)** Sankey diagram of primary and secondary metabolic pathways of vaginal microbiota, **(D)** Histogram of horizontal distribution of secondary and tertiary metabolic pathways of vaginal microbiota.

To assess the potential functional impact of PCOS on intestinal and vaginal microbiota, we conducted a predictive analysis of microbiota-related metabolic pathways using PICRUSt2 based on the KEGG database. The first-level metabolic function genes identified in both intestinal and vaginal microbiota predominantly belonged to categories such as Cellular Processes, Environmental Information Processing, Genetic Information Processing, Human Diseases, Glycan Pathways, and Metabolism, with the Metabolism pathway exhibiting the highest abundance (**Figures 6A, C**). We further identified the top 20 secondary metabolic pathways based on abundance. In the intestinal microbiota, potential functional genes were primarily associated with Lipid Metabolism, Metabolism of Other Amino Acids, Glycan Biosynthesis and Metabolism, Replication and Repair, and Translation (**Figure 6B**). Meanwhile, in the vaginal microbiota, potential functional genes were mainly related to Metabolism of Terpenoids and Polyketides, Metabolism of Other Amino Acids, Amino Acid Metabolism, Glycan Biosynthesis and Metabolism, and Lipid Metabolism (**Figure 6D**).

### 3.3 Correlation analysis of intestinal and vaginal microbiota with hormones and body weight in PCOS mice

**Figure 7:**
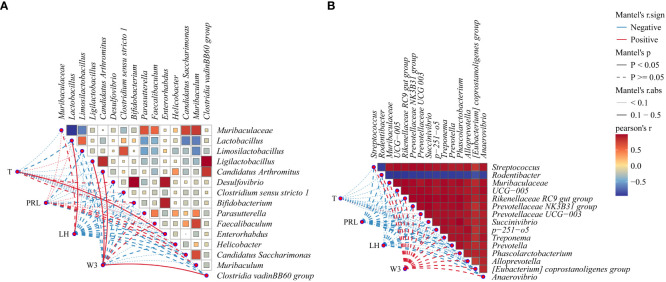
Correlation network diagram: **(A)** Correlation heatmap between intestinal microbiota and body weight (W3), T, PRL, and LH, **(B)** Correlation heatmap between vaginal microbiota and W3, T, PRL, and LH. Red indicates a positive correlation, and blue indicates a negative correlation. The solid line denotes *P* < 0.05, while the dashed line denotes *P* > 0.05. The R-value signifies the correlation strength: very weak (0.00-0.19), weak (0.20-0.39), moderate (0.40-0.59), strong (0.60-0.79), and very strong (0.80-1.00).

To examine the correlation between microbiota changes and PCOS mouse parameters, we conducted Pearson correlation analyses on third-week weight (W3), T, LH, and PRL using top 15 bacteria in intestinal and vaginal microbiota. **Figure 7A** highlights significant positive correlations: T with *Enterorhabdus* (R = 0.26, *P* = 0.031), LH with *Lactobacillus* (R = 0.47, *P* = 0.032). W3 showed positive correlations with *Ligilactobacillus* (R = 0.42, *P* = 0.002), *Candidatus Arthromitus* (R = 0.39, *P* = 0.029), and *Clostridia vadin*BB60 group (R = 0.34, *P* = 0.04). The results of the present study showed a significant increase in body weight and a significant increase in T in DHEA-modeled mice, confirming that hyperandrogenism and obesity interact with each other to influence the course of PCOS. A very strong negative correlation was observed between *Muribaculaceae* and *Lactobacillus*. *Desulfovibrio* demonstrated a very strong positive correlation with *Bifidobacterium* and *Enterorhabdus*, with *Bifidobacterium* exhibiting a very strong positive correlation with *Enterorhabdus*. The results of this study suggest that the interaction between hyperandrogen and obesity affects the course of PCOS. In **Figure 7B**, although correlations between factors and vaginal microbiota were not significant (*P* > 0.05), the primary results indicated negative associations of T, LH, and PRL with vaginal microbiota, while W3 displayed a positive correlation (*P* > 0.05). Notably, *Rodentibacter* had a very strong negative association with other bacteria in the top 15 relative abundance of vaginal microbiota, while all bacteria except *Rodentibacter* exhibited a very strong positive association.

## 4 Discussion

### 4.1 Hormonal imbalances and obesity in PCOS mice

HA is the most common and prominent diagnostic component of PCOS, and excess androgens induce accumulation of abdominal fat, resulting in visceral adiposity (Delitala et al., 2017). Thus, HA is usually closely associated with obesity. When androgens are in excess, adipocytes are enriched in large numbers thereby undergoing inflammation and apoptosis, releasing adipokines, affecting metabolism and sex steroid secretion, and leading to chronic low-grade inflammation and insulin resistance, and abdominal obesity and insulin resistance stimulate ovarian and adrenal androgen production, and may further increase abdominal obesity and inflammation, thus creating a vicious cycle (Spritzer et al., 2015). HA is assessed by both clinical features and/or biochemical markers, with elevated T in biochemical markers being the basis of diagnostic significance. The main detectors of ovulatory disorders are PRL and LH, which manifest as hyperprolactinemia and LH deficiency, and ovarian polycystic changes are the key and consistent features of polycystic ovaries (Homburg, 2008; Caldwell et al., 2014). The results of the present study showed a significant increase in body weight, PRL and T in modeled mice, a significant decrease in LH, the vaginal smears were in the intermotility phase, and the sections showed the presence of a definite polycystic lesion. Thus, intraperitoneal injection with DHEA was able to successfully replicate the PCOS mouse model with HA, impaired ovulation and ovarian polycystic changes.

### 4.2 Microbiota imbalances in PCOS mice

#### 4.2.1 Intestinal microbiota imbalances in PCOS mice

The microbiota and its metabolites are involved in the pathogenesis of obesity, insulin resistance, and impaired ovarian function by interacting with estrogens, androgens, insulin, and other hormones, modulating intestinal barrier function and influencing peripheral tissue physiology and metabolic function (Qi et al., 2021). Acidobacteriota is a bacterium that is widespread in the ecosystem and has been found to be associated with the seafloor sulfur cycle, with fewer studies on the regulatory mechanisms of the organism (Flieder et al., 2021). Desulfobacterota is a Gram-negative bacterium that is metabolized in the intestinal to produce hydrogen sulfide (H_2_S), which is toxic to the intestinal epithelial apparatus and leads to gastrointestinal disorders (Nguyen et al., 2023; Yang et al., 2023). Desulfobacterota and Acidobacteriota were found to be elevated in the CP group, which may be involved in the intestinal sulfur cycle and damage the intestinal mucosa through increased involvement with H_2_S-producing harmful bacteria.

*Limosilactobacillu* and *Lactobacillus* are a group of Gram-positive bacteria under the family Lactobacillaceae of the thick-walled phylum. Their ability to enhance the physical barrier of the mucus layer to improve immunity, their ability to produce lactic acid to maintain a weakly acidic environment as a major metabolic end product of carbohydrate fermentation (Petrova et al., 2015; Li C. et al., 2023), and modulation of Muribaculaceae abundance may ameliorate metabolic disorders by altering metabolism and butyrate production, and modulation of antibody and anti-inflammatory cytokine production to ameliorate inflammation (Hwang et al., 2023). In the present study, we found that the intestinal microbiota of *Muribaculaceae*, *Limosilactobacillus* and *Lactobacillus* were reduced in the CP group of mice. The CP group may contribute to the process of PCOS by reducing beneficial bacteria in the intestine, decreasing immunity in the mucus layer, and disrupting the acidic environment of the vagina leading to dysbiosis.

#### 4.2.2 Vaginal microbiota imbalances in PCOS mice

The vaginal microbiome is a niche in the human body composed mainly of *Lactobacillus*, capable of producing lactic acid from glycogen and responsible for acidifying the vaginal environment (Buchta, 2018).Vaginal microbiota imbalance is associated with a variety of factors such as disease, hormonal changes, stress, lifestyle, etc. Vaginal microbiota imbalance is characterized by a decrease in lactic acid bacteria and an increase in anaerobic microorganisms in the vaginal cavity (Miko and Barakonyi, 2023; Tuniyazi and Zhang, 2023). F/B values in the intestines and breast milk negatively correlate with body weight gain and obesity index (Chavoya-Guardado et al., 2022; Baek et al., 2023). This study showed that after modeling, vaginal microbiota imbalance F/B values decreased, body weight increased, and PCOS model mice replicated successfully. We therefore hypothesized that PCOS induces changes in the vaginal microbiota of mice and that weight gain may be associated with a decreased F/B ratio.

*Streptococcus* is a common gram-positive group of pyogenic cocci, of which pathogenic streptococci can cause a wide range of purulent inflammatory and hypersensitivity diseases in humans (Brouwer et al., 2023). It has been found that *Streptococcus* may influence the pathology of PCOS by affecting the female reproductive endocrine environment (Lu et al., 2021). *Muribaculaceae*, belonging to the phylum Mycobacterium, is a group of beneficial bacteria whose increased abundance may contribute to lipid metabolism disorders and alleviate obesity (Zhou et al., 2020). The relative abundance of the intestinal mucosal species *Muribaculaceae*, *Turicibacter*, and *Parasutterella* was significantly higher in female mice than in males, providing a better representation of the relationship between sexual dimorphism and sex hormones (Wu et al., 2022). In this study, it was found that *Streptococcus* and *Muribaculaceae* were increased in VP group. Therefore, with the increase of *Streptococcus* and the change of *Muribaculaceae* related to sex hormones, vaginal microbiota may participate in the regulation of sex hormones and affect the outcome of PCOS.

### 4.3 Microbiota-hormone disorder interactions in PCOS mice

Studies have shown that the relative abundance of *Enterorhabdus* in intestinal microbiota is related to glycolipid metabolism (Huang et al., 2023; Lahtinen et al., 2023), and *Enterorhabdus* abundance in PCOS mice is positively correlated with T level and blood glucose (Li Y. et al., 2023). Interferon may affect the expression level of important metabolites in uterine tissue by reducing the abundance of *Enterorhabdus* in intestinal microbiota, thus alleviating the injury of endometritis in mice (Xue et al., 2023). *Lactobacillus* has a protective effect on the ovaries, restoring luteinizing hormone, follicle-stimulating hormone, and testosterone levels, upregating short-chain fatty acid levels, and improving intestinal microbiota disorders (He et al., 2020). *Lactobacillus* transplantation and administration can improve the symptoms of PCOS, which is conducive to the treatment of PCOS (Guo et al., 2016; Siddiqui et al., 2022). *Candidatus Arthromitus* is involved in body glucose metabolism and is positively correlated with body weight (Fu et al., 2022; Zhao et al., 2022). Our study found that T was significantly positively correlated with *Enterorhabdus*, LH was significantly positively correlated with *Lactobacillus*, and body weight was significantly positively correlated with *Ligilactobacillus*, *Candidatus Arthromitus*. Therefore, intestinal microbiota may affect the process of PCOS by regulating the hormone metabolism of ovary, regulating T and LH, and improving *Enterorhabdus* and *Lactobacillus* may regulate the body weight of PCOS mice. Modulating the composition of the intestinal microbiota may be a therapeutic approach to controlling PCOS.

*Rodentibacter* is an opportunistic pathogen associated with promoters such as immune deficiencies, and its colonization does not usually result in clinical disease (Benga et al., 2019). But when the body has a disease, it may participate in the progression of the disease. It was found that gastrointestinal *Rodentibacter* and *Romboutsia* were negatively correlated with lung development in rats with intrauterine growth delay (Yang et al., 2022). The relative abundance of *Rodentibacter* in bronchoalveolar lavage fluid of mice with acute lung injury was positively correlated with the expression of interleukin-1β, interleukin-6, and tumor necrosis factor-α genes. Changing the abundance of *Rodentibacter* can alleviate acute lung injury (Jin et al., 2022). Our study found a significant negative correlation between *Rodentibacter* and vaginal microbiota, and that changes in the abundance of *Rodentibacter* may be involved in inflammatory response, regulation of lung development and angiogenesis. Therefore, we speculate that *Rodentibacter* may influence the course of the disease by participating in inflammation and growth and development in PCOS mice.

## 5 Conclusion

In conclusion, this study revealed changes in the gut and vaginal microbiota of PCOS mice, mainly consisting of reduced bacterial diversity, elevated Desulfobacterota, and decreased beneficial bacteria. Microbiota-hormone interactions revealed correlations between specific taxa and glycolipid metabolism, hormonal levels, and inflammation. *Enterorhabdus* and *Lactobacillus* emerged as key players in hormonal regulation. These findings emphasize the potential of regulating intestinal microbiota for PCOS management, indicating potential roles in obesity, insulin resistance, and impaired ovarian function, providing holistic insights into metabolic and reproductive health.

## Data availability statement

The datasets presented in this study can be found in online repositories. The names of the repository/repositories and accession number(s) can be found below: https://www.ncbi.nlm.nih.gov/, PRJNA1045307.

## Ethics statement

The animal study was approved by the Animal Ethics and Welfare Committee of the Hunan University of Chinese Medicine. The study was conducted in accordance with the local legislation and institutional requirements.

## Author contributions

XY: Data curation, Funding acquisition, Methodology, Resources, Supervision, Visualization, Writing – original draft, Writing – review & editing. XL: Conceptualization, Data curation, Formal Analysis, Methodology, Validation, Writing – review & editing. HY: Conceptualization, Funding acquisition, Investigation, Resources, Software, Visualization, Writing – review & editing.
